# A text-mining approach to obtain detailed treatment information from free-text fields in population-based cancer registries: A study of non-small cell lung cancer in California

**DOI:** 10.1371/journal.pone.0212454

**Published:** 2019-02-22

**Authors:** Frances B. Maguire, Cyllene R. Morris, Arti Parikh-Patel, Rosemary D. Cress, Theresa H. M. Keegan, Chin-Shang Li, Patrick S. Lin, Kenneth W. Kizer

**Affiliations:** 1 California Cancer Reporting and Epidemiologic Surveillance Program, Institute for Population Health Improvement, University of California Davis Health, Sacramento, California, United States of America; 2 University of California Davis, Graduate Group in Epidemiology, Davis, California, United States of America; 3 Department of Public Health Sciences, University of California Davis, Davis, California, United States of America; 4 Center for Oncology Hematology Outcomes Research and Training (COHORT) and Division of Hematology and Oncology, University of California Davis School of Medicine, Sacramento, California, United States of America; 5 School of Nursing, The State University of New York, University at Buffalo, Buffalo, New York, United States of America; 6 Department of Emergency Medicine, University of California Davis School of Medicine, Sacramento, California, United States of America; 7 Betty Irene Moore School of Nursing, University of California Davis, Sacramento, California, United States of America; Centro per lo Studio e la Prevenzione Oncologica, ITALY

## Abstract

**Background:**

Population-based cancer registries have treatment information for all patients making them an excellent resource for population-level monitoring. However, specific treatment details, such as drug names, are contained in a free-text format that is difficult to process and summarize. We assessed the accuracy and efficiency of a text-mining algorithm to identify systemic treatments for lung cancer from free-text fields in the California Cancer Registry.

**Methods:**

The algorithm used Perl regular expressions in SAS 9.4 to search for treatments in 24,845 free-text records associated with 17,310 patients in California diagnosed with stage IV non-small cell lung cancer between 2012 and 2014. Our algorithm categorized treatments into six groups that align with National Comprehensive Cancer Network guidelines. We compared results to a manual review (gold standard) of the same records.

**Results:**

Percent agreement ranged from 91.1% to 99.4%. Ranges for other measures were 0.71–0.92 (Kappa), 74.3%-97.3% (sensitivity), 92.4%-99.8% (specificity), 60.4%-96.4% (positive predictive value), and 92.9%-99.9% (negative predictive value). The text-mining algorithm used one-sixth of the time required for manual review.

**Conclusion:**

SAS-based text mining of free-text data can accurately detect systemic treatments administered to patients and save considerable time compared to manual review, maximizing the utility of the extant information in population-based cancer registries for comparative effectiveness research.

## Introduction

Population-based cancer registries contain information about treatment utilization and patient outcomes. Details about first-line systemic treatments are collected, mostly from electronic medical records, but only required standard data fields are coded [1]. Thus, much of the granular treatment information, such as drug names and regimens, is left uncoded in unstructured free-text fields. Because extracting and summarizing information from free-text fields through manual review is cumbersome and time consuming, this data source is infrequently used. However, evaluating survival outcomes by specific treatment type among all patients in a state cancer registry extends knowledge about the effectiveness of drug regimens reported in clinical trials to patient types usually ineligible for such trials (eg the elderly[2] and infirm[3]). In addition, treatment disparities by source of health insurance, race/ethnicity, socioeconomic status, and other determinants can be identified and addressed.

Several methods exist to facilitate the processing of text fields in health care. Extraction of information from text fields can be accomplished with natural language processing (NLP) and text mining. NLP is a complex computer-based extraction process that applies rule-based algorithms to combinations of terms, using linguistics and statistical methods to convert free text into a structured format [4, 5]. It has been used in a number of studies to extract clinically relevant information from electronic medical records [6–9]. It can be used in conjunction with machine learning to automate text evaluation [10, 11]. However, NLP and machine learning involve end-user development, customization, and ongoing support services from collaborators with expertise which can be costly [12]. Text mining includes a broad set of computerized techniques that allow for word and phrase matching [13, 14]. SAS software, widely used in data analyses, has text identification capabilities that can match words and patterns [15, 16]. It has been used to detect keywords in electronic health records to identify health conditions and to evaluate completeness of records [17–19].

We hypothesized that a SAS-based text-mining system could accurately detect specific treatment information from unstructured text fields in California Cancer Registry (CCR) data and substantially reduce the amount of time required for manual review. We tested this hypothesis with a categorization of systemic treatments utilized for patients with advanced-stage non-small cell lung cancer (NSCLC).The identification of specific advanced-stage NSCLC systemic treatments is of particular interest, given the dramatic changes observed over the past two decades with the introduction of targeted therapies and immunotherapies. Multiple systemic treatment options exist for NSCLC patients with stage IV disease. Patients can receive standard chemotherapy with platinum or non-platinum agents, bevacizumab (a vascular endothelial growth factor inhibitor) combined with other chemotherapy drugs, targeted therapy with tyrosine kinase inhibitors (TKIs), or immune checkpoint inhibitors, depending on tumor histology and biomarker status [20]. In this rapidly changing landscape, surveillance of systemic therapy utilization at the population level can provide insight into dissemination of new treatments and outcomes among all patient types. However, population-level studies are limited, partly due to the lack of a structured data source on NSCLC treatments. Previous studies have been restricted to particular drug regimens, specific age groups, and certain hospital types, or been done in non-U.S. communities [21–28].

Leveraging existing data collected by cancer registries in text fields with an efficient text-mining process could make routine use of these data feasible. The aims of this study were to (*1*) develop a SAS-based text mining algorithm to identify first-line systemic treatments among patients with stage IV NSCLC recorded in free-text fields in the CCR and (*2*) compare results obtained through text mining with those obtained through manual review of the same text fields to determine the algorithm accuracy.

## Methods

### Study population

We identified patients in the CCR age twenty years or older with first primary, stage IV NSCLC diagnosed from 2012 to 2014. The CCR is a population-based cancer surveillance system that includes all incident cancer diagnoses in California since 1988 with information on tumor characteristics, treatment, patient demographics, and annual follow-up for vital status. It collects incidence reports on more than 160,000 cases of cancer diagnosed annually in California. Data are collected through a network of regional registries, which are part of the National Cancer Institute’s Surveillance, Epidemiology and End Results program [1, 29–31].

We used the International Classification of Diseases for Oncology, 3^rd^ edition (ICD-O-3), World Health Organization site recode 2008 definition [32], to select individual lung cancer patients. We used the 2015 World Health Organization classification of lung tumors [33] to select the histologic types that comprise NSCLC. Excluded from analysis were autopsy only cases, death certificate only cases, and other values for sex (other, transsexual/transgender, not otherwise specified). Stage at diagnosis was assigned using the American Joint Committee on Cancer 7^th^ edition staging system rules [1, 34].

This study received an exempt determination from the University of California, Davis IRB.

### First-line systemic treatment groups

First-line systemic treatment was defined as the initial systemic or oral chemotherapy administered. First-line treatment is reported to the CCR by each treating facility where the patient was seen and is contained in free-text fields. If more than one treatment was reported for the patient, dates were used to determine the initial treatment.

The treatment text fields were first manually assessed by one reviewer who read the text and grouped treatments into six clinically meaningful categories that align with National Comprehensive Cancer Network treatment guidelines for the diagnosis years used in this study[20]. The six groups were as follows: 1) platinum doublets (any platinum chemotherapy in combination with another chemotherapy drug, excluding pemetrexed and bevacizumab); 2) pemetrexed-based combinations (pemetrexed alone or combined with a platinum agent); 3) bevacizumab-based combinations (bevacizumab alone or combined with platinum chemotherapy or another chemotherapeutic drug excluding pemetrexed; 4) pemetrexed plus bevacizumab-based combinations (used together or with a platinum agent); 5) single agent (platinum or nonplatinum); 6) TKIs. Patients with no treatment and unknown treatment were categorized into a seventh and eighth group.

If the text fields were blank or non-informative, then treatment was categorized as unknown. Treatment was categorized as ‘none’ only when there was indication that none was given such as ‘patient refused treatment’, ‘patient opted for hospice instead of treatment’, ‘no treatment given’, or ‘patient died before any treatment given’. The results from the manual review were used as the gold standard comparison for the text mining results.

### Algorithm

**Fig 1:**
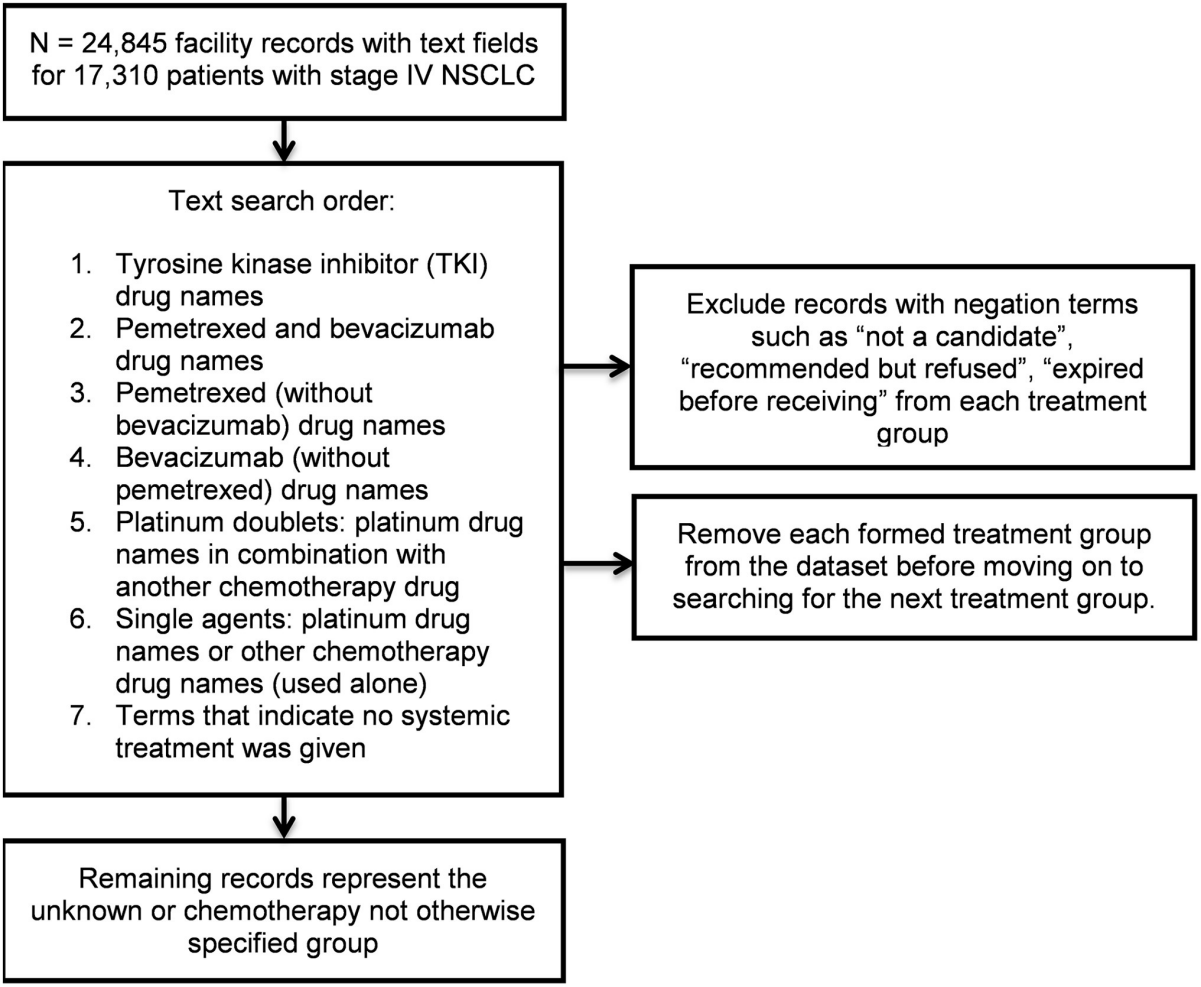
Text string search order for SAS-based text mining of non-small cell lung cancer (NSCLC) first-line systemic treatments in 17,310 patients diagnosed with stage IV disease, 2012–2104, California.

The same dataset assessed by manual review was evaluated using Perl regular expressions in SAS 9.4 software [16]. We matched records to each of the treatment groups by identifying drug names. Using the parsing capability of Perl regular expressions, we systematically searched for drugs one treatment group at a time starting with TKIs, then pemetrexed plus bevacizumab-based combinations, pemetrexed-based combinations, bevacizumab-based combinations, platinum doublets, and single agents (Fig 1). The search order moved from the groups with the fewest and most specific drugs to the groups with broader categories of drugs. We categorized records into the established groups by searching for the drug names associated with each group. In matching drug name search terms, we accounted for abbreviations, capitalization, brand names, and misspellings. This process involved some trial and error, with visual review of the matched and unmatched records after each treatment group search. Visual review of unmatched records revealed common misspellings and abbreviations. We also accounted for negation such as “not a candidate for…”, “recommended… but refused”, “expired before receiving…”. Once the algorithm was developed, the errors (false positives, false negatives) were not revised. For a complete list of search terms see S1 Table. After identifying records belonging in a treatment group, we removed these categorized records from the remaining records and then searched for drug names in the next treatment group. This was done for each of the six treatment groups. Next, search terms indicating that no treatment was given were used to identify the no systemic treatment group. The records remaining after removing all matched records for treatment groups and untreated patients were assigned to the unknown systemic treatment group.

### Analysis

The text mining assessment of systemic treatments was compared with the manual review findings (gold standard) for each of the six treatment groups, the no systemic treatment group, and the unknown group. Agreement was assessed with percent agreement and the kappa statistic. Percent agreement was calculated by dividing the true positives and true negatives reported by each method by the total number of patients in the sample. Kappa measures the proportion of agreement between methods after removing any chance agreement. For kappa, values of 0.61–0.80 are considered good and scores of 0.81–1.00 are considered excellent [35, 36]. In addition, sensitivity, specificity, positive predictive value (PPV), negative predictive value (NPV), false positives, false negatives, and total error were computed for each treatment group. Sensitivity measures the proportion of treated people who are correctly identified as treated, while specificity measures the proportion of untreated people who are correctly identified as untreated. PPV measures the proportion of people who test positive for treatment who actually were treated, while NPV measures the proportion of people who test negative for treatment who actually were untreated. False positives represent the number of untreated people incorrectly identified as having received treatment while false negatives represent the number of treated people incorrectly identified as not receiving treatment. Total error represents the total number of false positives and false negatives within each treatment group. Results are presented as percentages (%) and associated 95% confidence intervals (CI).

## Results

A dataset consisting of 24,845 free-text treatment records associated with 17,310 patients diagnosed 2012 to 2014 with stage IV NSCLC was evaluated manually and by SAS-based text mining. Specific treatment information was found for 78% of the patients. Percent agreement between text mining and manual review ranged from 91.1% to 99.4% (Table 1). Agreement was 98.1% (95% CI: 97.8%, 98.3%) for platinum doublets, 98.3% (95% CI 98.1%, 98.5%) for pemetrexed-based regimens, 99.4% (95% CI: 99.3%, 99.5%) for bevacizumab-based regimens, 99.2% (95% CI: 99.1%, 99.4%) for pemetrexed plus bevacizumab-based regimens, 98.7% (95% CI: 98.5%, 98.9%) for single agents, 97.7% (95% CI: 97.4%, 97.9%) for TKIs, 91.1% (95% CI: 90.7%, 91.5%) for no systemic treatment, and 91.6% (95% CI: 91.2%, 92.0%) for unknown treatment.

**Table 1:** Agreement of treatment between the SAS text mining algorithm and manual review among stage IV non-small cell lung cancer patients (n = 17, 310), 2012–2014, California.

SAS text mining		Manual Review						
		Yes	No	Total	Agreement		Kappa	
		n (% of total)	n (% of total)	n (% of total)	%	95% CI	Kappa	95% CI
Platinum doublets	Yes	2,442 (14.1)	90 (0.5)	2,532 (14.6)	98.1	97.8, 98.3	0.92	0.91, 0.93
	No	246 (1.4)	14,532 (84.0)	14,778 (85.4)				
	Total	2688 (15.5)	14,622 (84.5)	17,310				
Pemetrexed-based	Yes	1,974 (11.4)	159 (0.9)	2,133 (12.3)	98.3	98.1, 98.5	0.92	0.91, 0.93
	No	140 (0.8)	15,037 (86.9)	15,177 (87.7)				
	Total	2,114 (12.2)	15,196 (87.8)	17,310				
Bevacizumab-based	Yes	467 (2.7)	35 (0.2)	502 (2.9)	99.4	99.3, 99.5	0.90	0.88, 0.92
	No	63 (0.4)	16745 (96.7)	16808 (97.1)				
	Total	530 (3.1)	16780 (96.9)	17,310				
Pemetrexed and bevacizumab	Yes	618 (3.6)	114 (0.6)	732 (4.2)	99.2	99.1, 99.4	0.90	0.88, 0.92
	No	17 (0.1)	16561 (95.7)	16578 (95.8)				
	Total	635 (3.7)	16675 (96.3)	17,310				
Single agents	Yes	288 (1.7)	189 (1.1)	477 (2.8)	98.7	98.5, 98.7	0.71	0.68, 0.75
	No	37 (0.2)	16796 (97.0)	16833 (97.2)				
	Total	325 (1.9)	16985 (98.1)	17,310				
Tyrosine kinase inhibitors	Yes	1599 (9.2)	287 (1.7)	1886 (10.9)	97.7	97.4, 97.9	0.88	0.86, 0.89
	No	117 (0.7)	15307 (88.4)	15424 (89.1)				
	Total	1716 (9.9)	15594 (90.1)	17,310				
No systemic treatment	Yes	4,844 (28.0)	895 (5.2)	5,739 (33.2)	91.1	90.7, 91.5	0.80	0.78, 0.81
	No	642 (3.7)	10,929 (63.1)	11,571 (66.8)				
	Total	5,486 (31.7)	11,824 (68.3)	17,310				
Unknown systemic treatment	Yes	2,836 (16.4)	473 (2.7)	3,309 (19.1)	91.6	91.2, 92.0	0.74	0.73, 0.76
	No	981 (5.7)	13,020 (75.2)	14,001 (80.9)				
	Total	3,817 (22.1)	13,493 (77.9)	17,310				

Abbreviations: CI, confidence interval

Kappa values ranged from 0.71 to 0.92 (Table 1). Kappa was 0.92 (95% CI: 0.91, 0.93) for platinum doublets, 0.92 (95% CI: 0.91, 0.93) for pemetrexed-based regimens, 0.90 (95% CI: 0.88, 0.92) for bevacizumab-based regimens, 0.90 (95% CI: 0.88, 0.92) for pemetrexed plus bevacizumab-based regimens, 0.71 (95% CI: 0.68, 0.75) for single agents, 0.88 (95% CI: 0.86, 0.89) for TKIs, 0.80 (95% CI: 0.78, 0.81) for no systemic treatment, and 0.74 (95% CI: 0.73, 0.76) for unknown treatment.

Sensitivity, specificity, PPV, NPV are shown in Table 2. Pemetrexed plus bevacizumab-based regimens had the highest sensitivity (97.3%, 95% CI: 95.7%, 98.4%) while unknown treatment had the lowest (74.3%, 95% CI: 72.9%, 75.7%). Specificity ranged from 92.4% (no systemic treatment) to 99.8% (bevacizumab regimens) for all treatment groups. Single agents had the lowest PPV (60.4%, 95% CI: 56.8%, 63.8%) while platinum doublets had the highest (96.4%, 95% CI: 95.6%, 97.1%). NPV ranged from 92.9% (unknown treatment) to 99.9% (pemetrexed plus bevacizumab regimens).

**Table 2:** Sensitivity, secificity, PPV, and NPV of treatment identified with SAS-based text mining for stage IV non-small cell lung cancer patients (n = 17,310), 2012–2014, California.

	Sensitivity		Specificity		PPV		NPV	
Treatment Group	%	95% CI	%	95% CI	%	95% CI	%	95% CI
Platinum doublets	90.0	89.7, 91.9	99.4	99.2, 99.5	96.4	95.6, 97.1	98.3	98.1, 98.5
Pemetrexed-based regimens	93.4	92.2, 94.4	98.9	98.7, 99.1	92.5	91.4, 93.6	99.1	98.9, 99.2
Bevacizumab-based regimens	88.1	85.1, 90.7	99.8	99.7, 99.9	93.0	90.5, 94.9	99.6	99.5, 99.7
Pemetrexed and bevacizumab regimens	97.3	95.7, 98.4	99.3	99.1, 99.4	84.4	81.8, 86.6	99.9	99.8, 99.9
Single agents	88.6	84.6, 91.8	98.9	98.7, 99.0	60.4	56.8, 63.8	99.8	99.7, 99.8
Tyrosine kinase inhibitors	93.2	91.9, 94.3	98.2	97.9, 98.4	84.8	83.2, 86.2	99.2	99.1, 99.4
No systemic treatment	88.3	87.4, 89.1	92.4	91.9, 92.9	84.4	83.6, 85.2	94.5	94.1, 94.8
Unknown systemic treatment	74.3	72.9, 75.7	96.5	96.2, 96.8	85.7	84.6, 86.8	92.9	92.6, 93.3

Abbreviations: CI, confidence interval; NPV, negative predictive value; PPV, positive predictive value

Text mining errors for each treatment group are shown in Table 3. The no systemic treatment group had the most false positives (895, 5.2%) while the unknown treatment group had the most false negatives (981, 5.7%). Overall, the no systemic treatment group had the greatest total number of errors (1,537, 8.9%) followed by the unknown treatment group (1,454, 8.4%), TKIs (404, 2.3%), platinum doublets (336, 1.9%), pemetrexed-based regimens (299, 1.7%), single agents (226, 1.3%), pemetrexed plus bevacizumab-based regimens (131, 0.8%) and bevacizumab-based regimens (98, 0.6%).

**Table 3:** False positives, false negatives, and total errors for treatment identified with SAS text mining algorithm among stage IV non-small cell lung cancer patients (n = 17,310), 2012–2014, California.

	False Positives	False Negatives	Total Errors
Treatment Group	n (%)	n (%)	n (%)
Platinum doublets	90 (0.5)	246 (1.4)	336 (1.9)
Pemetrexed-based regimens	159 (0.9)	140 (0.8)	299 (1.7)
Bevacizumab-based regimens	35 (0.2)	63 (0.4)	98 (0.6)
Pemetrexed and bevacizumab regimens	114 (0.7)	17 (0.1)	131 (0.8)
Single agents	189 (1.1)	37 (0.2)	226 (1.3)
Tyrosine kinase inhibitors	287 (1.7)	117 (0.7)	404 (2.3)
No systemic treatment	895 (5.2)	642 (3.7)	1537 (8.9)
Unknown systemic treatment	473 (2.7)	981 (5.7)	1454 (8.4)

Percentages (%) represent percent of total

Time spent on manual review versus SAS-based text mining differed greatly. Manual review of the 24,845 records associated with the 17,310 patients took roughly 332 hours of the reviewer’s time at a rate of approximately 75 records an hour. Analysis of the same records with SAS-based text mining using Perl regular expressions, including programming time, took approximately 50 hours of the reviewer’s time.

## Discussion

In this study, systemic treatments were detected in unstructured free-text records for 17,310 patients using SAS-based Perl regular expressions with a high level of accuracy. Specific treatment information was found for 78% of patients. Percent agreement between the SAS-based text mining and the manual review varied by treatment group but was high for all groups (91.1% to 99.4%). Similarly, the kappa statistic showed good to excellent agreement for all groups (0.71–0.92) [35, 36]. Other studies have found similarly high concordance using SAS-based text mining. Percent agreement between manual review and SAS-based text mining was 96.6% for detection of follow-up appointments from discharge records while sensitivity, specificity, PPV, and NPV exceeded 94% for SAS-based detection of primary and recurrent cancers from electronic pathology reports [17, 18].

In our study, other measures of agreement showed more variation. Specificity (92.4%-99.8%) and NPV (92.9%-99.9%) were high for all groups, findings likely influenced by the large percentage of untreated patients (32%) in this study. The lower sensitivity (74.3%-97.3%) and PPV (60.4%-96.4%) estimates we observed resulted from false positives and false negatives. In particular, the low PPV for single agents is a consequence of the high number of false positives for this group. Isolating single agents administered as first-line treatment from records that list multiple treatments discussed or given over time proved difficult.

NLP and machine learning systems are becoming widely used and have reported successes in summarizing free text as well. Studies report that specially developed NLP and machine learning systems have correctly identified 92% of breast cancer recurrences, 96.8% of breast cancer cases, 84% of critical limb ischemia events, and 87% of cancer cases from electronic clinical notes or surgical pathology reports [6, 10, 37, 38]. Their ability to understand language and learn from experience make them powerful tools to explore free text in health records. However, NLP and machine learning require a level of expertise that research groups usually do not have on staff. The text mining presented in this study can be accomplished by researchers who have basic SAS programming skills.

**Fig 2:**
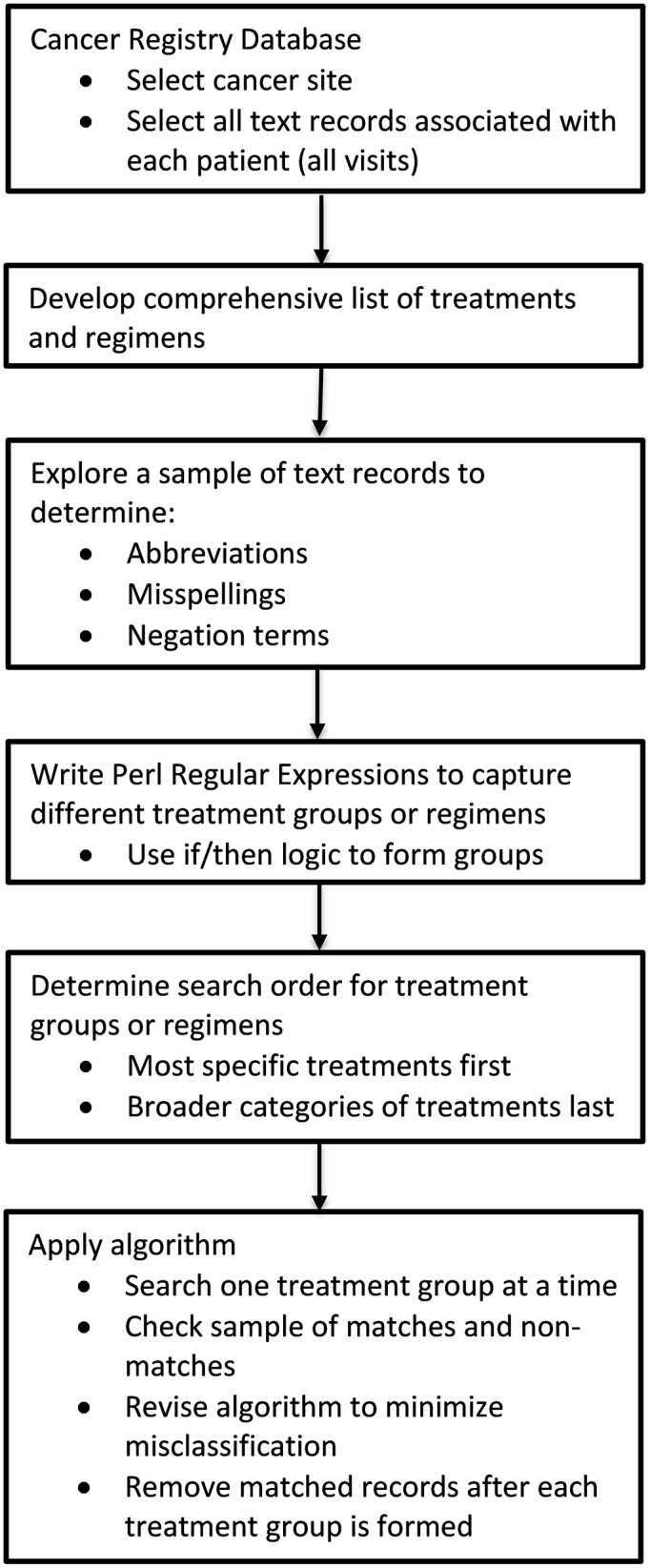
Process diagram for developing SAS-based text mining algorithm to summarize treatment information.

In addition to categorizing treatments with a high level of accuracy, our SAS-based text mining algorithm was easy to develop and enormously time saving compared to manual abstraction. Developing the algorithm (including programming) and applying it to the dataset used one-sixth of the time required for manual review of the same data. The same concepts used to create the algorithm for this study can be applied to other studies investigating treatments in free text. These include compiling a list of search terms, manually reviewing samples of the dataset to investigate abbreviations, misspellings, and negation terms, determining an order to search for groups and eliminate records, and investigating matches and non-matches throughout the process (Fig 2). Our findings suggest that future efforts to extract and summarize treatment information from CCR data or other cancer registries have the potential to be completed relatively quickly with a high degree of confidence in the accuracy of the results and without the need for a lengthy comprehensive manual review process.

Some limitations were present. Because only one reviewer for the manual abstraction of the treatment text fields was used, we were unable to measure the reliability of the reviewer. Additionally, systemic treatments were unknown for 22% of the patients. It is likely that many of these patients did not receive systemic treatment. Studies have documented that approximately half of patients with advanced stage lung cancer do not receive systemic treatment [28, 39]. However, there was variability in the unknowns by SEER (Surveillance, Epidemiology and End Results Program) reporting region and by hospital National Cancer Institute designation suggesting that some under-reporting occurred.

SAS-based text mining has some limitations as well. Although concordance was high, some misclassification occurred, highlighting a shortcoming of text mining; negation and uncertainty are not accounted for when matching on words or word fragments. To counteract this, a collection of regular expressions that identify potential negation (“not a candidate for…”, “refused…”) and treatment uncertainty (“recommended…unknown if given) were used, but a fair number of false positives were still present. Additionally, many text fields list multiple treatment options discussed or various treatments received over the course of time, making it difficult to identify the first-line treatment. This resulted in both false positives and false negatives. Furthermore, although the six treatment groups in this study are mutually exclusive, some of the same drugs are used in more than one group (ie. pemetrexed, platinum agents) making the treatment group classifications with text mining challenging and resulting in some misclassification. To minimize text mining misclassification, samples of treatment groups should be manually reviewed and compared to text mining results. Where misclassification is high, more effort should be put into search terms, negation terms, and misspellings.

Furthermore, there are license fees associated with the use of SAS. While there are some open source software packages capable of performing text mining, NLP, and machine learning tasks, SAS is commonly used in academic research settings [15].

This study had several strengths. It used robust population-based data that included patients treated across a large spectrum of facilities, which increases the generalizability of the findings. In addition, our algorithm can be customized and applied to other cancer types and to other text fields that contain details about radiation treatment, surgery, laboratory tests, and pathology findings that are not summarized in the data, thus expanding on the information that is currently available in the CCR. Further studies exploring other cancer sites and their associated text fields in the CCR are warranted.

## Conclusion

In conclusion, we found SAS-based text mining to be accurate and efficient in summarizing systemic lung cancer treatment text fields. A thorough understanding of the data, a comprehensive list of search terms, and manual testing are essential to its successful implementation. However, mining unstructured free-text fields greatly decreases the time and resources needed to review and summarize these fields manually, maximizing the utility of the extant information, and making routine use of these text fields feasible for comparative effectiveness research.

## Supporting information

S1 Table Text mining search strings and SAS regular expressions used to categorize treatment groups. (DOCX)
