# Supplementary material for: A text-mining approach to obtain detailed treatment information from free-text fields in population-based cancer registries: A study of non-small cell lung cancer in California
Source: PLoS One. 2019 Feb 22;14(2):e0212454. doi: 10.1371/journal.pone.0212454 (PMC6386345; doi:10.1371/journal.pone.0212454)
Supplement: S1 Table — (DOCX) [file pone.0212454.s001.docx]

S1 Table. Text mining search strings and SAS regular expressions used to categorize treatment groups

S1 Table. Text mining search strings and SAS regular expressions used to categorize treatment groups, continued
